# Selective Serotonin Reuptake Inhibitor Use Is Associated with Right Ventricular Structure and Function: The MESA-Right Ventricle Study

**DOI:** 10.1371/journal.pone.0030480

**Published:** 2012-02-17

**Authors:** Corey E. Ventetuolo, R. Graham Barr, David A. Bluemke, Aditya Jain, Joseph A. C. Delaney, W. Gregory Hundley, Joao A. C. Lima, Steven M. Kawut

**Affiliations:** 1 Department of Medicine, Alpert Medical School of Brown University, Providence, Rhode Island, United States of America; 2 Department of Medicine, College of Physicians and Surgeons, and the Departments of Epidemiology and Biostatistics, Mailman School of Public Health, Columbia University, New York, New York, United States of America; 3 Radiology and Imaging Sciences, National Institutes of Health/Clinical Center, National Institute for Biomedical Imaging and Bioengineering, Bethesda, Maryland, United States of America; 4 Department of Medicine, Johns Hopkins University School of Medicine, Baltimore, Maryland, United States of America; 5 Department of Pharmaceutical Outcomes and Policy, College of Pharmacy, University of Florida, Gainesville, Florida, United States of America; 6 Department of Internal Medicine/Cardiology, Wake Forest University School of Medicine, Winston-Salem, North Carolina, United States of America; 7 Department of Medicine, Penn Cardiovascular Institute, and the Center for Clinical Epidemiology and Biostatistics, Perelman School of Medicine, University of Pennsylvania, Philadelphia, Pennsylvania, United States of America; Ohio State University, United States of America

## Abstract

**Purpose:**

Serotonin and the serotonin transporter have been implicated in the development of pulmonary hypertension (PH). Selective serotonin reuptake inhibitors (SSRIs) may have a role in PH treatment, but the effects of SSRI use on right ventricular (RV) structure and function are unknown. We hypothesized that SSRI use would be associated with RV morphology in a large cohort without cardiovascular disease (N = 4114).

**Methods:**

SSRI use was determined by medication inventory during the Multi-Ethnic Study of Atherosclerosis baseline examination. RV measures were assessed via cardiac magnetic resonance imaging. The cross-sectional relationship between SSRI use and each RV measure was assessed using multivariable linear regression; analyses for RV mass and end-diastolic volume (RVEDV) were stratified by sex.

**Results:**

After adjustment for multiple covariates including depression and left ventricular measures, SSRI use was associated with larger RV stroke volume (RVSV) (2.75 mL, 95% confidence interval [CI] 0.48–5.02 mL, p = 0.02). Among men only, SSRI use was associated with greater RV mass (1.08 g, 95% CI 0.19–1.97 g, p = 0.02) and larger RVEDV (7.71 mL, 95% 3.02–12.40 mL, p = 0.001). SSRI use may have been associated with larger RVEDV among women and larger RV end-systolic volume in both sexes.

**Conclusions:**

SSRI use was associated with higher RVSV in cardiovascular disease-free individuals and, among men, greater RV mass and larger RVEDV. The effects of SSRI use in patients with (or at risk for) RV dysfunction and the role of sex in modifying this relationship warrant further study.

## Introduction

Serotonin, a potent vasoconstrictor, and the serotonin transporter (5-HTT) have mitogenic effects on pulmonary artery smooth muscle cells (PA-SMCs) [1]. Serotonin has been implicated in the development of pulmonary arterial hypertension (PAH) since anorexigens (which activate 5-HTT) were noted to increase the risk of PAH [2]. Some patients with PAH may have elevated levels of serotonin which persist beyond treatment [3], [4], [5]. 5-HTT overexpression leads to the development of pulmonary hypertension (PH) and PA-SMC hyperplasia, and 5-HTT polymorphisms have been associated with disease development [1], [6], [7], [8]. Selective serotonin reuptake inhibitors (SSRIs) inhibit 5-HTT, making them appealing therapies for study in pulmonary vascular disease.

SSRIs protect against pulmonary vascular changes and right ventricle (RV) hypertrophy in animal models of pulmonary vascular disease [9], [10], [11]. Human PA-SMCs demonstrate attenuated growth when exposed to SSRIs [1], [12]. We have observed a possible lower risk of death in PAH patients treated with SSRIs compared to those not treated [13]. More recently, Shah et al. found SSRI use was associated with both a decreased incidence of PAH and reduced mortality in patients with PAH [14]. A randomized clinical trial to evaluate the efficacy of escitalopram in patients with PAH was recently completed (NCT00190333).

While there is compelling evidence that SSRIs may be a promising therapy in pulmonary vascular disease, the effects of SSRI use on RV function, an important determinant of outcome in PH and PAH, are unknown. Studying the impact of potential PH therapies on RV structure and function in individuals without clinical cardiovascular disease (or in those with subclinical disease) may inform the study of novel therapeutics in patients with pulmonary vascular disease and RV failure. We examined the cross-sectional association of SSRI use with RV structure and function assessed by cardiac magnetic resonance imaging (MRI) in a large cohort without clinical cardiovascular disease. We hypothesized that SSRI use would be associated with larger RV stroke volume (RVSV) and higher RV ejection fraction (RVEF) and lower RV mass, end-diastolic volume (RVEDV), and end-systolic volume (RVESV).

## Methods

### Study Sample

The protocols of the Multi-Ethnic Study of Atherosclerosis (MESA) and studies described herein were approved by the Institutional Review Boards of all institutions (Columbia University, New York; Johns Hopkins University, Baltimore; Northwestern University, Chicago; University of California, Los Angeles; University of Minnesota, Twin Cities; Wake Forest University, Winston-Salem) and the National Heart Lung and Blood Institute. Written informed consent was obtained from all participants. MESA is a multicenter prospective cohort study to investigate subclinical cardiovascular disease in Caucasians, African-Americans, Hispanics, and Chinese [15]. In 2000–2002, MESA recruited 6,814 participants aged 45–84 years old from six U.S. communities: Forsyth County, NC; Northern Manhattan and the Bronx, NY; Baltimore City and Baltimore County, MD; St. Paul, MN; Chicago, IL; and Los Angeles, CA. Exclusion criteria included clinical cardiovascular disease, weight >300 lbs, pregnancy, or impediment to long-term participation. The presence of clinical cardiovascular disease was determined at screening by questionnaire. Participants were excluded if they answered “yes” to having been diagnosed by a physician with heart attack, stroke, transient ischemic attack, heart failure, angina, current atrial fibrillation, and/or to having undergone any prior cardiovascular procedure. The MESA-Right Ventricle Study measured RV morphology in 4204 participants with interpretable MRIs. MESA-Right Ventricle participants were sampled without regard to demographics, anthropometrics, or other clinical variables.

### Cardiac Magnetic Resonance Imaging Measures

The cardiac MRI protocol has been described elsewhere [16]. All imaging was performed on 1.5 T magnets with a 4-element phased-array surface coil positioned anteriorly and posteriorly and electrocardiographic gating. Imaging consisted of fast gradient echo cine images with temporal resolution ≤50 ms.

Methods for interpretation of left ventricle (LV) and RV parameters have been previously reported [16], [17]. Briefly, RV image analysis was performed by two independent analysts on Windows workstations using QMASS software (v4.2, Medis, the Netherlands). The endocardial and epicardial borders of the RV were traced manually on short axis cine images at the end-diastolic and end-systolic phase. Papillary muscles and trabeculae were included in the RV volumes and excluded from RV mass [18]. RVEDV and RVESV were calculated using Simpson's rule by summation of areas on each slice multiplied by the sum of slice thickness and image gap. RV mass was determined at end-diastole as the difference between end-diastolic epicardial and endocardial volumes multiplied by the specific gravity of the heart (1.05 g/cm^3^) [16]. RVSV was calculated by subtracting RVESV from RVEDV. RVEF was calculated by dividing RVSV by RVEDV. The intra-reader intraclass correlation coefficient (ICC) from random, blinded re-reads of 229 scans for RV mass was 0.94 and for 230 scans was 0.99, 0.95, and 0.89 for RVEDV, RVESV, and RVEF, respectively. The intra-reader ICC was 0.96 for RVSV. The inter-reader ICC from random, blinded re-reads of 240 scans for RV mass, RVEDV, RVESV, and RVEF was 0.89, 0.96, 0.94 and 0.80, respectively. The inter-reader ICC for RVSV was 0.93.

### Selective Serotonin Reuptake Inhibitor Use

A validated medication inventory was used to assess SSRI use during the baseline exam [19]. Participants were asked to bring all containers for medications used during the two weeks prior to the baseline visit. Interviewers transcribed all current medication names, strengths, and dosages from medication bottles. Participants were queried about actual medication intake and interviewers recorded the average number of pills taken. In circumstances where no medication was taken, the interviewer coded medication intake as “0”. Total duration of SSRI use (beyond the two weeks prior to the baseline visit) was not available. Fluoxetine, fluvoxamine, paroxetine, and sertraline were classified as high-affinity SSRIs (dissociation constant [K_d_]<1 nmol) and citalopram as a medium-affinity SSRI ([K_d_]>1 nmol) for sub-group analyses.

### Other Covariates

Race/ethnicity was self-reported during the baseline exam according to 2000 US Census criteria as race (Caucasian, African-American, etc) and ethnicity (Hispanic or non-Hispanic). Standard questionnaires were used to ascertain smoking status and level of education. Medication use was ascertained by medication inventory [19]. A small number of participants taking anorexigens (N = 13) were excluded from the study sample. Height was measured to the nearest 0.1 cm with the participant in stocking feet and weight was measured to the nearest pound with the participant in light clothing using a balanced scale. Resting blood pressure was measured using the Dinamap Monitor PRO 100 (Critikon, Tampa, FL) automated oscillometric device. Hypertension was defined as systolic blood pressure ≥140 mm Hg, diastolic blood pressure ≥90 mm Hg or current use of anti-hypertension medication. Fasting blood samples were drawn and sent to a central laboratory for measurement of lipids. The presence of depressive symptoms was assessed during the baseline examination using the Center for Epidemiologic Studies Depression Scale (CES-D) [20]. Depression was defined as a CES-D score ≥16 points [20].

### Statistical Analysis

Continuous variables were expressed as means and standard deviations. Categorical variables were expressed as %. Independent sample *t* tests were used to compare continuous variables and chi-square tests were used to compare categorical variables in SSRI users and non-users. Multivariate linear regression was used to assess the relationship of SSRI use with each RV parameter. Limited models included age, sex, race/ethnicity, height, weight, and level of education. Adjustment for height and weight avoided the assumptions made in indexing the RV measures to parameters of body size (e.g., body surface area), while accounting for differences in body size between participants.

We selected covariates which were possibly related to either SSRI use or RV morphology. We retained covariates in the models if their inclusion resulted in a change (>10%) in the effect estimate for SSRI use and any of the RV measures. Adjusted models included age, sex, race/ethnicity, height, weight, level of education, depression (CES-D ≥16), smoking (status and pack-years), hypertension, systolic and diastolic blood pressure, cholesterol, low-density lipoprotein levels, triglycerides, and statin use. Possible covariates that were considered but not adjusted for (based on this criterion) included waist circumference, diabetes mellitus, impaired glucose tolerance, antihypertensive medication use, high density lipoprotein levels, serum sex hormone levels, and intentional exercise. While depression (CES-D ≥16 points) did not significantly confound the relationship between SSRI use and RV measures by this criterion, this covariate was forced into the adjusted models. Finally, respective LV measures were added to the adjusted models to account for the contribution of LV abnormalities to RV changes (e.g., increased LV mass causing pulmonary venous hypertension leading to increased RV mass), to account for body size differences, and to examine RV-specific associations. RVSV was not adjusted for LV stroke volume considering the significant inter-dependence of these measures. Statistical significance was defined as P<0.05. Analyses were performed using STATA 10.0 (StataCorp, College Station, TX).

## Results

**Figure 1:**
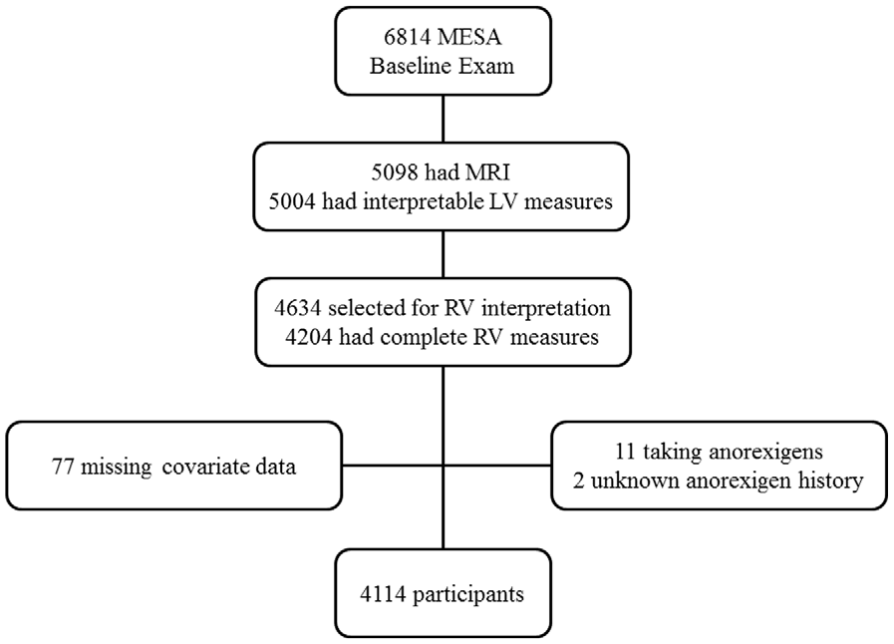
Study sample. MESA: Multi-Ethnic Study of Atherosclerosis; MRI: magnetic resonance imaging; LV: left ventricle; RV: right ventricle.

MESA enrolled 6814 participants of whom 5098 had available cardiac MRI and 5004 had scans interpretable for LV measures (Figure 1). Of these, 4634 were selected, and 4204 (the *a priori* sample size for MESA-Right Ventricle) were successfully interpreted for RV measures. We excluded participants with a self-reported (N = 11) or unknown (N = 2) history of anorexigen use and those with missing covariate data (N = 77). The final study sample consisted of 4114 participants.

The characteristics of the study sample and excluded participants are shown in Table 1. Overall, the study sample was similar to those excluded. In the study sample, 203 (4.9%) were using SSRIs. Among SSRI users, 182 (89.7%) used high-affinity agents. Characteristics of SSRI users and non-users are shown in Table 2. SSRI users were more likely to be female, Caucasian, and better educated compared to non-users. The prevalence of hypertension and smoking was similar between the two groups, while SSRI users had higher mean cholesterol and triglyceride levels, were more likely to be taking statins, and were more likely to have been treated for diabetes. Not surprisingly, SSRI users had higher mean CES-D scores and more commonly had depression. There were significant interactions between SSRI use and sex in terms of RV mass (p for interaction = 0.02) and RVEDV (p for interaction = 0.01). Analyses for these parameters are shown stratified by sex. Table 3 compares characteristics of the study sample by sex. Women tended to be less well educated, never smokers, and had a higher prevalence of depression. While men tended to be taller and weigh more, body mass index (BMI) was similar between the sexes.

**Table 1:** Characteristics of the study sample and of those participants excluded.

		Study Sample	Excluded
Number		4114	2700
**Demographics**			
Age, years		61.5±10.1	63.2±10.4
Male, %		47.6	46.5
Race/ethnicity, %	Caucasian	39.4	37.1
	African-American	26.2	30.2
	Hispanic	21.9	22.0
	Chinese	12.5	10.7
Education, %	<High school	16.3	20.7
	High school	18.4	17.9
	< College, (>high school)	28.5	28.6
	≥College	36.8	32.8
**Anthropometrics**			
Height, cm		166.4±9.9	166.3±10.2
Weight, kg		77.5±16.2	80.8±18.8
Body mass index, kg/m^2^		27.9±5.0	29.1±6.1
**Comorbid Factors**			
Hypertension, %		42.9	48.0
Systolic blood pressure, mm Hg		125.5±21.0	128.3±22.1
Diastolic blood pressure, mm Hg		71.9±10.2	72.0±10.4
Diabetes mellitus, %	Normal	75.2	71.0
	Impaired fasting glucose	13.2	14.8
	Untreated diabetes	2.4	3.0
	Treated diabetes	9.2	11.3
Total cholesterol, mg/dl		194.4±35.0	193.8±36.8
Low-density lipoprotein, mg/dl		117.4±30.8	116.9±32.4
Triglycerides, mg/dl		130.8±84.9	132.8±94.5
Statin use, %		14.7	15.1
**Smoking Status**	Never-smoker, %	52.1	47.6
	Former smoker, %	35.4	38.5
	Current smoker, %	12.5	13.9
Pack years, among ever-smokers		10.9±22.8	12.2±21.4
**Depression and Antidepressant Use**			
Center for Epidemiologic Studies Depression Scale (CES-D), points		7.4±7.4	7.9±7.8
Depression (CES-D ≥16), %		12.4	14.9
Antidepressant use, %	Single agent, %	8.8	8.1
	Combination (>1 agent), %	0.3	0.1
Selective serotonin reuptake inhibitors, %		4.9	4.5
	Medium affinity*	0.5	0.8
	High affinity†	4.4	3.7
Selective-norepinephrine reuptake inhibitors, %		0.6	0.6
Tricyclic antidepressants, %		1.3	1.4
Other‡, %		2.0	1.6

Data shown as mean ± standard deviation or %. Percentages may not add to one because of rounding.

*Citalopram.

† Fluoxetine, fluvoxamine, paroxetine, and sertraline.

‡ Includes atypical (bupropion), monoamine oxidase inhibitors, tetracyclic (mirtazapine), and serotonin antagonist and reuptake inhibitor (trazodone and nefazodone) antidepressants.

**Table 2:** Characteristics of SSRI users and non-users included in the study sample.

		SSRI Users	SSRI Non-users	P value
Number		203	3911	
**Demographics**				
Age, years		60.4±9.9	61.6±10.1	0.11
Male, %		31.0	48.4	<0.001
Race/ethnicity, %				<0.001
	Caucasian	64.0	38.1	
	African-American	11.8	27.0	
	Hispanic	19.2	22.1	
	Chinese	4.9	12.9	
Education, %				0.06
	<High school	9.9	16.6	
	High school	17.7	18.5	
	< College, (> high school)	30.5	28.4	
	≥College	41.9	36.6	
**Anthropometrics**				
Height, cm		164.9±8.9	166.5±10.0	0.03
Weight, kg		77.2±15.6	77.5±16.2	0.76
Body mass index, kg/m^2^		28.3±5.2	27.8±5.0	0.20
**Comorbid Factors**				
Hypertension, %		42.9	42.9	1.00
Systolic blood pressure, mm Hg		124.7±19.8	125.5±21.1	0.57
Diastolic blood pressure, mm Hg		70.0±9.8	72.0±10.2	0.01
Diabetes mellitus, %				0.02
	Normal	77.8	75.0	
	Impaired fasting glucose	8.9	13.4	
	Untreated diabetes	0.5	2.5	
	Treated diabetes	12.8	9.0	
Total cholesterol, mg/dl		200.0±34.4	194.1±35.0	0.02
Low-density lipoprotein, mg/dl		118.1±31.0	117.4±30.8	0.73
Triglycerides, mg/dl		141.3±78.5	130.3±85.2	0.07
Statin use, %		22.2	14.3	<0.01
**Smoking Status**				0.33
	Never-smoker, %	47.3	52.4	
	Former smoker, %	39.9	35.2	
	Current smoker, %	12.8	12.5	
Pack years, among ever-smokers		13.7±23.2	10.8±22.7	0.07
**Depression**				
Center for Epidemiologic Studies Depression Scale (CES-D), points		10.8±9.5	7.2±7.3	<0.01
Depression (CES-D ≥16), %		24.1	11.8	<0.001
**RV measures**	RVSV, mL	87.9±21.2	86.8±20.6	0.46
	RVEF, %	71.0±6.0	70.4±6.5	0.22
	RV mass, g	21.1±4.5	21.0±4.5	0.91
	RVEDV, mL	124.4±31.1	124.1±30.9	0.91
	RVESV, mL	36.5±13.6	37.3±14.3	0.43

Data shown as mean ± standard deviation or %. Percentages may not add to one because of rounding.

**Table 3:** Characteristics of men and women included in the study sample.

		Men	Women
Number		1957	2157
**Demographics**			
Age, years		61.5±10.1	61.5±10.1
Race/ethnicity, %	Caucasian	38.6	40.1
	African-American	25.2	27.1
	Hispanic	23.5	20.6
	Chinese	12.8	12.2
Education, %	< High school	15.5	17.0
	High school	15.7	20.9
	< College, (> high school)	26.0	30.7
	≥ College	42.8	31.4
**Anthropometrics**			
Height, cm		173.4±7.7	160.0±7.0
Weight, kg		83.1±14.8	72.4±15.8
Body mass index, kg/m^2^		27.5±4.1	28.2±5.6
**Comorbid Factors**			
Hypertension, %		40.9	44.6
Systolic blood pressure, mm Hg		125.1±18.9	125.8±22.7
Diastolic blood pressure, mm Hg		74.9±9.3	69.1±10.2
Diabetes mellitus, %	Normal	71.6	78.4
	Impaired fasting glucose	15.7	10.9
	Untreated diabetes	2.9	2.0
	Treated diabetes	9.8	8.7
Total cholesterol, mg/dl		188.1±33.8	200.2±35.1
Low-density lipoprotein, mg/dl		116.9±30.4	117.8±31.2
Triglycerides, mg/dl		133.6±86.7	128.3±83.2
Statin use, %		13.8	15.5
Smoking Status	Never-smoker, %	42.6	60.8
	Former smoker, %	43.4	28.1
	Current smoker, %	14.1	11.1
Pack years, among ever-smokers		14.3±27.6	7.9±16.7
**Depression**			
Center for Epidemiologic Studies Depression Scale (CES-D), points		6.3±6.5	8.4±8.0
Depression (CES-D ≥16), %		8.0	16.3
**RV measures**	RVSV, %	95.9±20.7	78.6±16.7
	RVEF, %	68.2±6.2	72.5±6.0
	RV mass, g	23.1±4.4	19.2±3.6
	RVEDV, mL	140.9±29.7	108.9±23.2
	RVESV, mL	45.1±14.1	30.3±10.2

Data shown as mean ± standard deviation or %. Percentages may not add to one because of rounding.

### Selective Serotonin Reuptake Inhibitor Use

SSRI use was associated with a 2.75 mL (3.2%) higher RVSV after adjustment for all covariates (95% confidence interval [CI] 0.48–5.02 mL, p = 0.02) (Table 4). There was no association between SSRI use and RVEF. In men, SSRI use was associated with a 4.7% higher RV mass after adjustment for all covariates including LV mass (1.08 g, 95% CI 0.19–1.97 g, p = 0.02), but there was no association in women. Also, there was a stronger association between SSRI use and larger RVEDV (5.5% larger) among men than among women (1.7% larger). While not statistically significant, SSRI use may have been associated with larger RVESV after adjustment for LV end-systolic volume.

**Table 4:** Associations between SSRI use and RV measures in limited and adjusted models, stratified by sex for RV mass and RVEDV.

	Beta (95% CI)	P value	MenBeta (95% CI)	P value	WomenBeta (95%CI)	P value
**RVSV, mL**						
Limited*	2.54 (0.26–4.83)	0.03	–	–	–	–
Adjusted†	2.75 (0.48–5.02)	0.02	–	–	–	–
**RVEF, %**						
Limited	0.02 (−0.84–0.88)	0.97	–	–	–	–
Adjusted	−0.14 (−1.01–0.72)	0.74	–	–	–	–
Adjusted + LVEF	−0.23 (−1.03–0.57)	0.57	–	–	–	–
**RV mass, g**						
Limited	–	–	0.89 (−0.05–1.83)	0.06	0.05 (−0.45–0.54)	0.86
Adjusted	–	–	1.01 (0.06–1.96)	0.04	0.13 (−0.37–0.63)	0.61
Adjusted + LV mass	–	–	1.08 (0.19–1.97)	0.02	0.06 (−0.41–0.53)	0.80
**RVEDV, mL**						
Limited	–	–	4.99 (−1.30–11.28)	0.12	2.55 (−0.55–5.65)	0.11
Adjusted	–	–	5.80 (−0.54–12.14)	0.07	3.24 (0.14–6.34)	0.04
Adjusted + LVEDV	–	–	7.71 (3.02–12.40)	0.001	1.84 (−0.47–4.15)	0.12
**RVESV, mL**						
Limited	0.77 (−0.76–2.31)	0.32	–	–	–	–
Adjusted	1.15 (−0.39–2.70)	0.14	–	–	–	–
Adjusted + LVESV	1.21 (−1.18–2.59)	0.09	–	–	–	–

*Adjusted for age, sex, race/ethnicity, height, weight, and level of education.

† Adjusted for age, sex, race/ethnicity, height, weight, level of education, depression (Center for Epidemiologic Studies.

Depression-Scale (CES-D) ≥16), smoking (status and pack-years), hypertension, systolic and diastolic blood pressure, cholesterol, low-density lipoprotein levels, triglycerides, and statin use.

After limiting our analysis to those using high-affinity agents (N = 182, 89.7% of SSRI users), we found similar associations with RVSV and no association with RVEF (Table 5). As with the total study sample, there were significant interactions between SSRI use and sex for RV mass (p for interaction = 0.02) and RVEDV (p for interaction = 0.01). In men, high-affinity SSRI use was associated with greater RV mass even after adjustment for LV mass (1.30 g, 95% CI 0.35–2.24 g, p = 0.01), but there was no association in women. The association of high-affinity SSRI use with larger RVEDV was strong among men but was much weaker in women (and not present after adjustment for LV end-diastolic volume).

**Table 5:** Associations between high affinity‡ SSRI use and RV measures in limited and adjusted models, stratified by sex for RV mass and RVEDV.

	Beta (95% CI)	P value	MenBeta (95% CI)	P value	WomenBeta (95%CI)	P value
**RVSV, mL**						
Limited*	2.59 (0.18–4.99)	0.04	–	–	–	–
Adjusted†	2.85 (0.47–5.24)	0.02	–	–	–	–
**RVEF, %**						
Limited	−0.08 (−0.99–0.83)	0.86	–	–	–	–
Adjusted	−0.25 (−1.15–0.66)	0.60	–	–	–	–
Adjusted + LVEF	−0.26 (−1.09–0.58)	0.55	–	–	–	–
**RV mass, g**						
Limited	–	–	1.15 (0.14–2.17)	0.03	0.15 (−0.37–0.67)	0.57
Adjusted	–	–	1.21 (0.19–2.23)	0.02	0.25 (−0.27–0.77)	0.35
Adjusted + LV mass	–	–	1.30 (0.35–2.24)	0.01	0.20 (−0.30–0.69)	0.43
**RVEDV, mL**						
Limited	–	–	5.31 (−1.45–12.07)	0.12	2.83 (−0.41–6.07)	0.09
Adjusted	–	–	5.94 (−0.81–12.70)	0.09	3.67 (0.43–6.90)	0.03
Adjusted + LVEDV	–	–	7.78 (2.77–12.78)	<0.01	1.80 (−0.62–4.21)	0.14
**RVESV, mL**						
Limited	0.88 (−0.73–2.49)	0.29	–	–	–	–
Adjusted	1.28 (−0.33–2.90)	0.12	–	–	–	–
Adjusted + LVESV	1.19 (−0.26–2.64)	0.11	–	–	–	–

*Adjusted for age, sex, race/ethnicity, height, weight, and level of education.

† Adjusted for age, sex, race/ethnicity, height, weight, level of education, depression (Center for Epidemiologic Studies Depression-Scale (CES-D) ≥16), smoking (status and pack-years), hypertension, systolic and diastolic blood pressure, cholesterol, low-density lipoprotein levels, triglycerides, and statin use.

‡ Fluoxetine, fluvoxamine, paroxetine, and sertraline.

## Discussion

SSRI use was associated with higher RVSV in men and women and with greater RV mass in men only. SSRI use may have been associated with larger RVESV in both sexes and was more strongly associated with larger RVEDV in men than in women. There was no association between SSRI use and RVEF. To our knowledge, this is the only study of SSRI use and RV morphology in humans.

Increased stroke volume and end-diastolic volume without a change in ejection fraction is seen in the LV in athletes, and even in the RV in MESA participants who report higher levels of exercise [21], [22]. This suggests that the SSRI-associated changes may be adaptive. Animal models suggest that RV adaptation to exercise may not only outstrip LV changes, but that RV eccentric hypertrophy leads to an increase in capillary networks and oxygen transport, implying a compensatory response [23], [24]. While ventricular response to pressure loading has been well-described in the LV, in pulmonary vascular disease the spectrum of RV changes (from adaptive to maladaptive) is less well understood [25]. In animals with moderate PH, exercise results in enhanced capillarization (implying compensation) whereas in those with progressive disease increased RV diameter and wall stress develops (implying maladaptation), albeit with a similar degree of hypertrophy in both moderate and progressive PH [26]. Several small observational and a single randomized trial have examined changes in RV morphology with therapy in PAH patients at advanced stages of disease (i.e., predominantly World Health Organization functional class II/III) and shown decrements in RV mass and volumes with improved systolic function [27], [28], [29]. While we postulate that the SSRI-related associations with RV mass and volumes seen here may be adaptive in health or in subclinical disease, the clinical impact of SSRI-related changes in patients with advanced pulmonary vascular disease is not known.

In our study, the association between SSRI use and greater RV mass (in men only) persisted after adjustment for LV mass, implying that 5-HTT inhibition may not only uniquely impact the RV but that these effects may vary by sex. Endurance training and subsequent ventricular hypertrophy have been correlated with increased aerobic capacity in athletes, and interestingly men may have a more pronounced response as compared to women [22], [30], [31]. In experimental PH, 5-HTT inhibition leads to reductions in pulmonary artery pressure and subsequent decreases in RV mass [10]. However, our observations suggest that in normal individuals presumably without significant pulmonary vascular disease, SSRI use is associated with increased RV mass. Although not feasible in a disease-free cohort of over 4000 individuals, hemodynamic data from right heart catheterization could have elucidated the association between SSRI use and pulmonary vascular function.

We have recently shown that serum sex hormone levels are associated with RV morphology in a sex-specific manner [32]. While sex hormones do not appear to confound the associations seen here, sex is known to play a role in serotonin-stimulated platelet aggregation and kinetics and 5-HTT is differentially regulated in men and women [33], [34], [35]. Additionally, RV structure and function vary by sex in both health and disease, with men having greater RV mass and volumes but lower RVEF than women, suggesting SSRIs may differentially impact morphology depending on sex [36], [37]. While adjustment for sex hormone levels did not change effect estimates in our study, it is certainly possible that hormones may underpin different morphologic changes in men and women.

Animal studies of acute and chronic left heart failure have implicated serotonin as an important regulator of cardiac hypertrophy [38], [39]. In young animals, knock-out of serotonin receptors results in dilated cardiomyopathy, suggesting an important role of serotonin in the developing heart. Receptor overexpression in adult animals, however, leads to maladaptive hypertrophy [40]. Epidemiologic studies suggest that the use of SSRIs in patients with ischemic heart disease may be beneficial [41], [42], [43], [44]. In a randomized clinical trial of SSRIs to treat patients with major depressive disorder and acute coronary syndrome, SSRI use did not affect LV ejection fraction, but did lower risk of adverse cardiovascular events, possibly by inhibiting platelet activation and preserving endothelial function [45], [46]. A follow-up clinical trial of sertraline in depressed patients with congestive heart failure (CHF) showed no difference in composite cardiovascular events between intervention and control arms [47]. A larger trial of escitalopram in CHF is on-going and includes sub-studies to investigate escitalopram's effects on platelet function and endothelial vasoreactivity [48].

The impact of serotonin and its pathways on RV structure and function has not been extensively studied. In animals, serotonin causes protein oxidation in the RV (but not in the LV) and treatment with SSRIs prevents RV hypertrophy and lowers RV weight in PH models [11], [49], [50], [51]. Treatment with SSRIs suppresses PA-SMC proliferation but also increases pulmonary arterial cellular apoptosis, which may or may not be beneficial in the RV [52]. While serotonin released from pulmonary endothelial cells stimulates growth of PA-SMCs, it is unknown how cardiac endothelial function and myocytes might be affected by these paracrine effects [12]. Finally, it is difficult to reconcile the potential benefit of SSRIs in PAH with the observational evidence that maternal SSRI use may increase the risk of persistent pulmonary hypertension of the newborn in infants [53].

Our study has several limitations. While the effect sizes seem small (ranging from 3.2% to 5.5%), they are comparable to those seen in the LV related to active smoking and diabetes mellitus and in the RV related to physical activity (e.g., a 7% increase in RVEDV and a 5% increase in RV mass with higher levels of physical activity), but more subtle than the associations we have shown in the RV related to obesity [54], [55], [56]. A few small studies have shown similar magnitudes of effect on RV tissue Doppler imaging [57], [58], [59]. In severe PAH, long-term intravenous epoprostenol improves RVSV by approximately 12% [28]. In a normal individual, similar or smaller differences (in absolute or relative terms) may therefore have important physiologic effects. As our study is observational, no conclusions can be drawn about a causal role of SSRI use in RV morphology. Confounding by indication is possible, although it is unlikely that SSRIs were differentially prescribed based on subclinical differences in RV structure and function. Certain covariates may have influenced both SSRI use and RV measures, leading to unmeasured or residual confounding. While recall bias could be present, the medication inventory method utilized has been validated and medication use was assessed before the RV data were available, making differential recall based on RV morphology unlikely [19]. Unfortunately, duration of SSRI use was not assessed but would have allowed for further quantification of the observed associations. Some participants could have been on long-term SSRIs, while others for only a short period of time (a minimum of two weeks). However, such misclassification would likely bias to the null (e.g., a participant taking an SSRI for a few months before the baseline study visit would be unlikely to experience RV effects, yet would still be classified as “exposed”).

We have shown that SSRI use is associated with higher RVSV, larger RVEDV and greater RV mass (in men) in cardiovascular disease-free participants. Studies of SSRIs in patients with or at risk for pulmonary vascular disease should include specific assessments of RV effects and sex may prove important in modifying this relationship.
